# Supplementary material for: Crotalphine Modulates Microglia M1/M2 Phenotypes and Induces Spinal Analgesia Mediated by Opioid-Cannabinoid Systems
Source: Int J Mol Sci. 2022 Sep 30;23(19):11571. doi: 10.3390/ijms231911571 (PMC9569646; doi:10.3390/ijms231911571)
Supplement: Supplementary file 1 [file ijms-23-11571-s001.zip › ijms-1820899-supplementary.pdf]

Supplementary

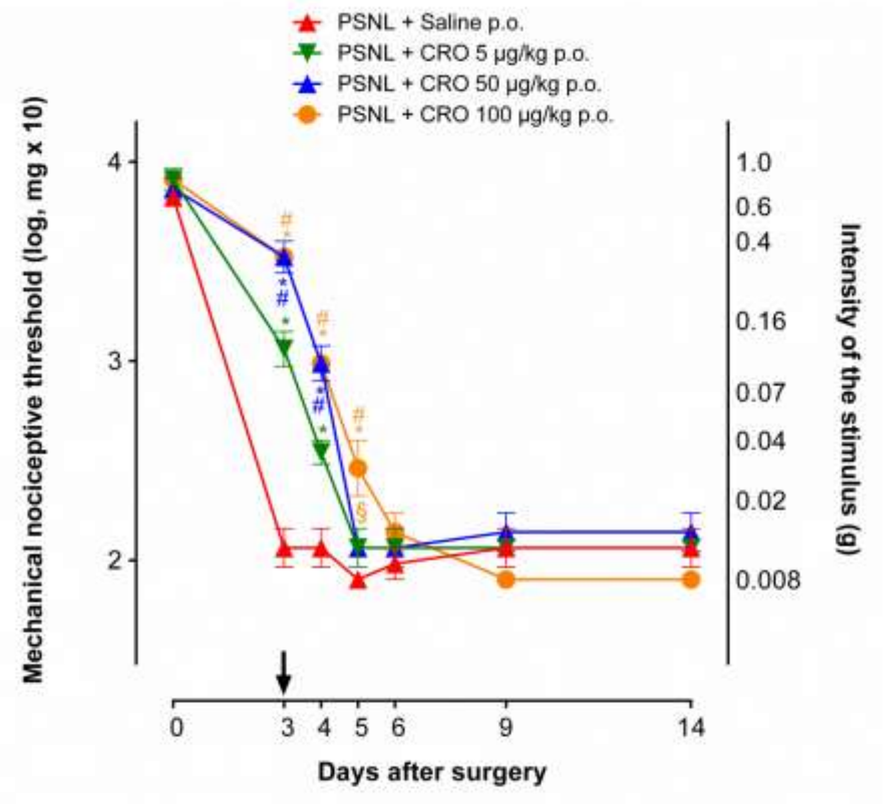

Supplementary figure S1. Analgesia induced by crotalphine in the acute phase does not prevent the development of chronic pain. The animals were treated on the 3rd day after PSNL with oral crotalphine (CRO), as indicated by the arrow. The nociceptive threshold was assessed before, one hour after, and on the days indicated the following treatment using von Frey filaments. The results are expressed by the mean of (± EPM) n = 5. * $p < 0.05$ indicates a statistically significant difference when compared to the PSNL + Saline group. # $p < 0.05$ indicates a statistically significant difference when compared to the PSNL + CRO group 5 µg / kg. § $p < 0.05$ indicates a statistically significant difference when compared to the PSNL + CRO 50 µg / kg group. The two-way ANOVA test was used followed by the Tukey test.

A

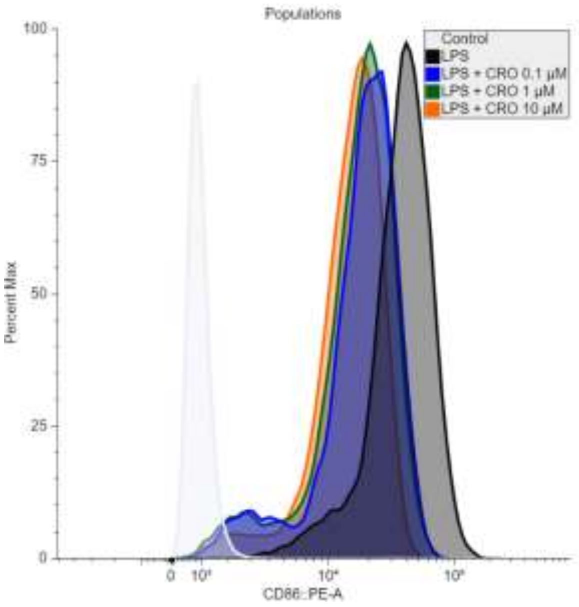

B

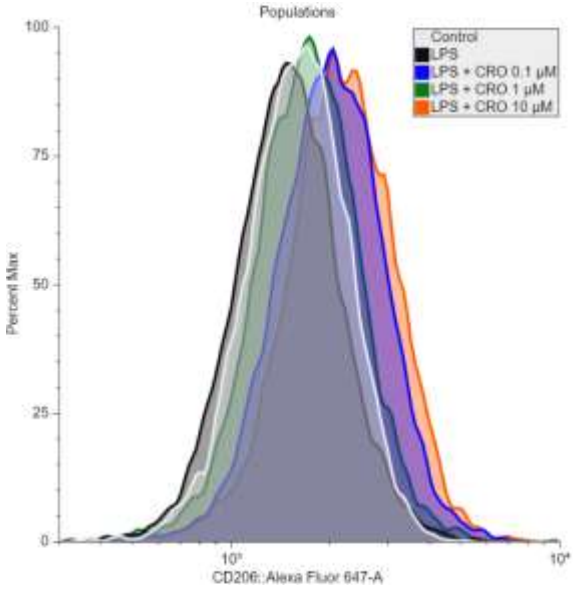

Supplementary figure S2. Representative histograms from the surface expression of the molecules CD86 and CD206 in BV-2 cells. BV-2 cells were incubated with LPS, LPS + CRO 0.1, LPS + CRO 1 or LPS + CRO 10 μM. The expression of CD86 (**A**) and CD206 (**B**) was assessed by flow cytometry.

Analyzing tool for synthetic crotalphine physiochemical properties prediction and blood-brain barrier penetrating potential

The physicochemical properties of crotalphine were analyzed using peptide synthesis and proteotypic peptide analyzing tool. These tolls are available at the Thermo Fisher Scientific website (https://www.thermofisher.com/br/en/home/life-science/protein-biology/peptides-proteins/custom-peptide-synthesis services/ peptide-analyzing-tool.html), and Expasy portal (http://web.expasy.org/protparam), which includes information about molecular weight, theoretical isoelectric point (pI), amino acid composition, atomic composition, extinction coefficient, estimated half-life, instability index, aliphatic index and grand average of hydropathicity (GRAVY). The blood-brain barrier penetrating potential was analyzed using the computational models BBPpredict [67] (http://i.uestc.edu.cn/BBPpredict/cgi-bin/BBPpredict.pl) and BBPpred (http://bbppred.xialab.info/).

Results

Crotalphine aliphatic index and grand average of hydropathicity (GRAVY), used to represent the hydrophobicity value of a peptide (hydropathy values of all the amino acids divided by the sequence length), were calculated using both analyzing tools, indicating a hydrophilic peptide (GRAVY= -1.31). The blood-brain barrier penetrating potential, analyzed by computational models, indicates that crotalphine is not predicted to easily penetrate the blood-brain barrier, with a probability of only 0.36 and 0.12.
